# Calcium overload-induced apoptosis in cancer cells: ER-mitochondria crosstalk and therapeutic implications

**DOI:** 10.1016/j.pscia.2026.100129

**Published:** 2026-06-22

**Authors:** Yucui Ding, Xinyu Liu, Jianyue Xue, Jianlong Fu, Sha Liu, Xiaopeng Xu, Xiang Jiao, Zhenyong Wu, Ganqiang Yang, Hongbo Wang, Peng Zhang

**Affiliations:** a School of Pharmacy, Key Laboratory of Molecular Pharmacology and Drug Evaluation (Yantai University), Ministry of Education, Collaborative Innovation Center of Advanced Drug Delivery System and Biotech Drugs in Universities of Shandong, Yantai University, Yantai, 264005, China; b Shandong Laboratory of Yantai Drug Discovery, Bohai Rim Advanced Research Institute for Drug Discovery, Yantai, Shandong, 264117, China

**Keywords:** Calcium overload, Endoplasmic reticulum stress, Mitochondrial dysfunction, Calcium-based nanocarriers, Anti-tumor therapy

## Abstract

Calcium overload exhibits significant anti-tumor potential by inducing abnormal intracellular Ca^2+^ accumulation, which disrupts mitochondrial and endoplasmic reticulum (ER) functions, thereby triggering apoptosis. However, its clinical application is currently hindered by challenges such as poor tumor-targeting capabilities, insufficient tumor accumulation, and incomplete mechanistic understanding. This review systematically analyzes the structural and functional coupling between the ER and mitochondria to elucidate the mechanisms of calcium overload-mediated cell death. We highlight how Ca^2+^ acts as a critical trigger to amplify mitochondria-associated ER stress, fostering a self-amplifying loop of crosstalk that initiates tumor cell death pathways. Furthermore, we summarize recent advances in targeted Ca^2+^ delivery using calcium-based nanocarriers combined with emerging modalities like sonodynamic therapy (SDT) and photothermal therapy (PTT), highlighting their synergistic antitumor potential. Compared with previous reviews, this work focuses on recent calcium-based nanosystems, sequential ER–mitochondria damage during Ca^2+^ overload, the MAM-associated IP3R–GRP75–VDAC1–MCU axis, and future strategies for tumor-targeted, TME-responsive, and multimodal synergistic therapy. By summarizing current research, this review aims to provide a prospective outlook for the novel anti-cancer therapies that target the disruption of intracellular Ca^2+^ homeostasis.

## Highlights

• Ca^2+^-overload and sequential organelle damage as a novel antitumor therapy.
• MAMs coupling role in orchestrating intracellular Ca^2+^ homeostasis.
• Nanosystems for precision Ca^2+^ regulation and oxidative stress amplification.
• Future prospects for intelligent targeting and multimodal synergistic therapy.

## Graphical abstract

**Figure:**
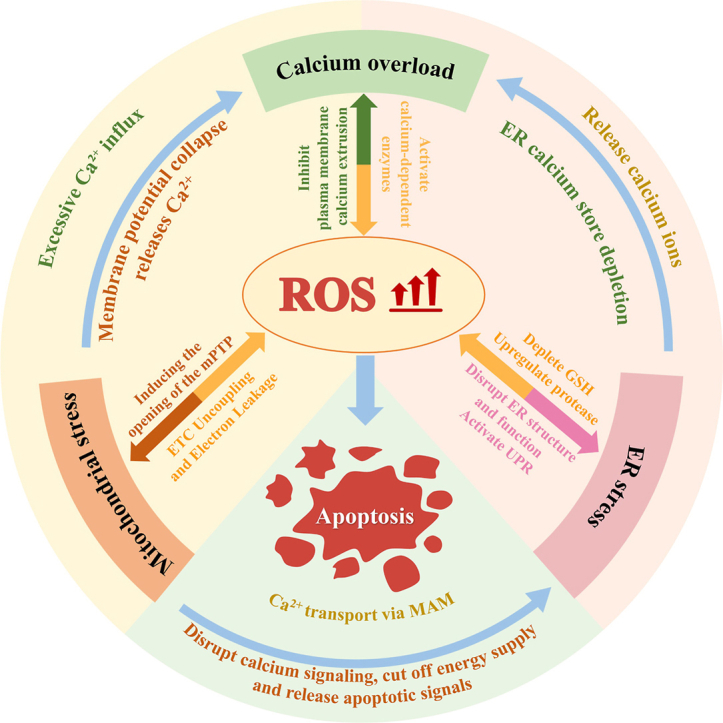

## 1 Introduction

Despite advances in molecular oncology and nanomedicine, cancer remains a leading cause of death globally, largely due to tumor heterogeneity and drug resistance [1,2]. Metabolic reprogramming in cancer cells creates an abnormal dependence on calcium (Ca^2+^) signaling to sustain proliferation, survival, and adaptation to microenvironmental stress [3,4]. Consequently, therapeutic strategies that specifically disrupt Ca^2+^ homeostasis to activate cell death pathways have emerged as a promising antitumor approach.

The endoplasmic reticulum (ER) and mitochondria, two central organelles for Ca^2+^ storage and energy metabolism, are physically and functionally coupled through dynamic membrane contact sites, particularly the mitochondria-associated ER membranes (MAMs) [5,6]. Excessive intracellular Ca^2+^ accumulation, commonly referred to as calcium overload, can induce ER stress, mitochondrial dysfunction, and multiple forms of cell death. Recent studies have leveraged this mechanism by developing Ca^2+^-based nanocarriers (e.g., CaCO_3_, CaO_2_) that release Ca^2+^ in the acidic tumor microenvironment (TME), often combined with photothermal or sonodynamic therapy to amplify oxidative stress [[7], [8], [9], [10]]. However, critical issues remain insufficiently addressed in existing literature: (i) the precise molecular hierarchy by which MAM-resident proteins translate Ca^2+^ signals into mitochondrial injury and cell-death execution; (ii) whether Ca^2+^-induced cell death are a conserved mechanism or exhibits tumor-type-specific dependence; and (iii) how to overcome off-target toxicity (especially cardiotoxicity and neurotoxicity), as well as poor tumor targeting, and insufficient biocompatibility of current nanocarriers for clinical translation.

To address these gaps, this review focuses the core MAM-associated signaling axis composed of IP3R, GRP75, VDAC1, and MCU to explain how ER–mitochondria Ca^2+^ transfer is coupled to the mitochondrial permeability transition pore (mPTP) opening, cytochrome *c* release, and caspase-dependent mitochondrial apoptosis. Ca^2+^-based nanocarriers are critically evaluated in terms of targeting efficiency, biocompatibility, and in vivo degradation, with additional discussion of tumor-specific Ca^2+^ intervention strategies, major clinical translation barriers, and potential solutions. By integrating molecular mechanisms, tumor specificity, and nanomedicine-based delivery strategies, this review provides a forward-looking framework for developing clinically viable Ca^2+^ overload-based antitumor therapy strategies.

## 2 ER-mitochondrial interaction

Rather than functioning as independent organelles, the ER and mitochondria are functionally connected via specialized MCSs, particularly the mitochondria-associated membranes (MAMs). This dynamic interface provides the structural foundation for rapid signal transduction and metabolic control, while acting as a critical connection for calcium homeostasis and apoptotic regulation [11,12].

### 2.1 Structural coupling between the ER and mitochondria

**Fig. 1:**
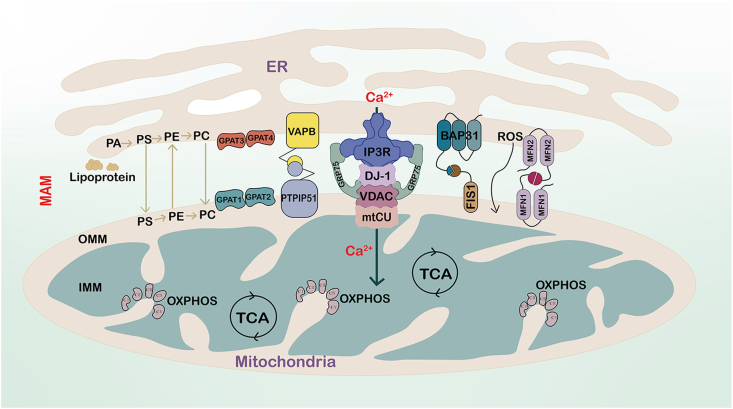
Schematic illustration of tethering complexes at the mitochondria-associated ER membrane (MAM), including VDAC-IP3R, MFN1/2, BAP31-FIS1, and VAPB-PTPIP51. Created by the authors using Adobe Illustrator.

While maintaining distinct functional environments, the ER and mitochondria are physically bridged by dynamic and reversible interfaces known as the ER-mitochondria contact sites (ERMCS). The specific membranous constituents involved in the ERMCS are referred to as the mitochondrial-associated endoplasmic reticulum membrane (MAM). Within this specialized microdomain, the membranes of the mitochondria and ER are only 10 – 25 nm [13]. This membrane interface is not just a simple contact zone but is characterized by numerous protein channels and signaling complexes (Fig. 1) [14]. These molecular connections play a key role in maintaining and regulating the balance of both the ER and mitochondria, controlling the transport of Ca^2+^ and enabling the direct synthesis and exchange of lipids between the ER and mitochondria [15,16].

Advances in proteomics have identified over a thousand proteins associated with the formation and function of the MAM [17,18]. Among these, certain tethering complexes are essential for preserving the structural integrity needed for signaling. For instance, the Vesicle-associated membrane protein-associated protein B (VAPB), localized on the ER membrane, interacts with the Tyrosine Phosphatase Interacting Protein-51 (PTPIP51) that on the outer mitochondrial membrane [19]. Similarly, the mitochondrial fusion proteins Mitofusin 1 (Mfn1) and Mitofusin 2 (Mfn2) establish crucial physical linkages. Mfn2, mainly found at the ERMCS, combines with mitochondrial Mfn1 to form hetero-oligomeric complexes. Disruption of these links, the cell signaling could be disrupted [20]. Such tight structural coupling enables the precise regulation of Ca^2+^, which is fundamental to cellular energy production and survival [21,22]. Notably, mitochondria exhibit marked sensitivity to fluctuations in Ca^2+^ concentration [23,24].

Under physiological conditions, the ER serves as the primary calcium reservoir, whereas mitochondria are not a major storage site for Ca^2+^ but take up the Ca^2+^ released from the ER [25,26]. The resting cytosolic Ca^2+^ concentration is generally maintained at approximately 50–100 nM. In contrast, upon ER release, the local Ca^2+^ concentration within the MAM microdomain can transiently reach to 100–300 μM, thereby creating a steep gradient that facilitates the rapid transfer of Ca^2+^ from the ER to the mitochondria [[27], [28], [29]]. The most functionally critical tethering complex for this calcium signaling includes the Inositol 1,4,5-Triphosphate Receptor (IP3R) on the ER and the Voltage-Dependent Anion Channel (VDAC) on the outer mitochondrial membrane [30,31]. These two channels are physically connected by the 75-kDa glucose-regulated protein (GRP75) forming the IP3R-GRP75-VDAC complex [32,33]. This complex forms a Ca^2+^ channel that links the ER and mitochondria, regulating Ca^2+^ flux to maintain organellar calcium balance and thereby modulate cellular function and apoptosis. Recent research has identified that the DJ-1 protein is a key part of the IP3R-Grp75-VDAC complex. The loss of DJ-1 leads to IP3R aggregation and prevents the formation of the IP3R3-Grp75-VDAC1 complex, thereby compromising ER-mitochondrial integrity. This precise regulation is crucial for mitochondria to receive enough calcium to produce ATP without exceeding the toxic thresholds that trigger cell death [34,35].

To further clarify the specific regulatory roles of MAM-associated proteins in calcium overload-induced apoptosis, the core ER–mitochondria calcium-transfer signaling axis is summarized. In this process, IP3R functions as the principal ER Ca^2+^ release channel in response to pro-apoptotic stimuli, while GRP75 acts as a molecular chaperone and physical tether that couples IP3R to VDAC, thereby facilitating efficient Ca^2+^ transfer from the ER to mitochondria. VDAC mediates Ca^2+^ passage across the outer mitochondrial membrane. Although MCU is not a MAM tethering protein, it serves as the key inner mitochondrial membrane uniporter responsible for Ca^2+^ entry into the mitochondrial matrix [36,37]. When mitochondrial matrix Ca^2+^ exceeds a critical threshold, sustained mPTP opening induces mitochondrial membrane depolarization, mitochondrial swelling, and outer membrane permeabilization, resulting in cytochrome *c* release and subsequent caspase-dependent apoptosis [38,39]. This process may be further amplified by ROS-mediated positive feedback, as mitochondrial Ca^2+^ overload and mPTP opening promote mitochondrial dysfunction and ROS accumulation, which can sensitize ER Ca^2+^ release channels such as IP3R, thereby reinforcing ER-to-mitochondria Ca^2+^ transfer and apoptotic signaling [40].

Beyond metabolic regulation, the ER–mitochondria axis also function as a critical checkpoint for cell fate by modulating apoptotic signaling [41]. Under conditions where cellular stress exceeds adaptive capacity, protein interactions within the MAM can convert calcium signals into pro-death signals [42]. This transformation involves specific protein couplers such as the Integrin B cell receptor-associated protein 31 (BAP31). In response to stress, BAP31 interacts with the pro-apoptotic p53 target Cell Death-Inducing p53 Target 1 (CDIP) to establish stress-responsive communication and facilitate ER stress-mediated apoptosis [43,44]. Furthermore, BAP31 also couples with the mitochondrial fission protein 1 homolog (Fis1), and the BAP31–Fis1 complex acts as a platform that recruits caspase-8 and promotes ER Ca^2+^ release, there by disrupting mitochondrial function and triggering calcium-dependent apoptosis [45,46].

### 2.2 Functional complementarity between the ER and mitochondria

#### 2.2.1 Lipid synthesis

The close structural collocation between the ER and mitochondria establishes a necessary physical platform for coordinated lipid metabolism [47]. While the ER is regarded as the primary locus for lipid biosynthesis, the MAM functions as the critical interface for lipid exchange and intermediate processing [48,49]. This functional coupling is exemplified by the distinct spatial segregation of glycerol-phosphate acyltransferases (GPATs), which GPAT3 and GPAT4 are localized to the ER and GPAT1 and GPAT2 reside on the outer mitochondrial membrane [50]. Hence, the efficiency of lipid synthesis depends on the functional integrity of the channeling complexes connecting these organelles rather than the isolated activity of a single compartment. For example, the specific enrichment of the yeast acyltransferase MLG1(a paralogue of PSI1) at the MAM interface is pivotal for driving the synthesis of phosphatidic acid [51]. Investigations indicate that the depletion of Mfn2 precipitates a loss of contact sites, thereby directly impairing the capacity for cholesterol synthesis [52]. These findings substantiate that the architectural integrity of the MAM is the indispensable prerequisite for the maintenance of cellular lipid homeostasis.

Indeed, the biosynthesis of functional lipids is rarely completed within the ER alone. Numerous lipid species must be translocated to the mitochondria via the MAM for essential enzymatic modification. A typical example is the generation of phosphatidylethanolamine (PE), this synthesis process requires the transfer of phosphatidylserine (PS) from the ER to the mitochondria through the MAM interface for catalytic conversion [53]. Similarly, sphingolipids such as ceramide, require MAM-mediated transport to the mitochondria to exert their downstream effects, particularly in regulating apoptosis [54]. By driving non-vesicular lipid transport and secondary conversions, the MAM acts as a critical “transit station” that defines the biological function of cellular lipids [55,56]. Beyond phospholipid metabolism, the MAM also plays a pivotal role in the transport and metabolic regulation of cholesterol. Research has identified that the MAM-resident Sigma-1 receptor directly affects the efficiency of cholesterol transport into the mitochondria by modulating the VDAC2 [57].

#### 2.2.2 Ca^2+^ translocation

**Fig. 2:**
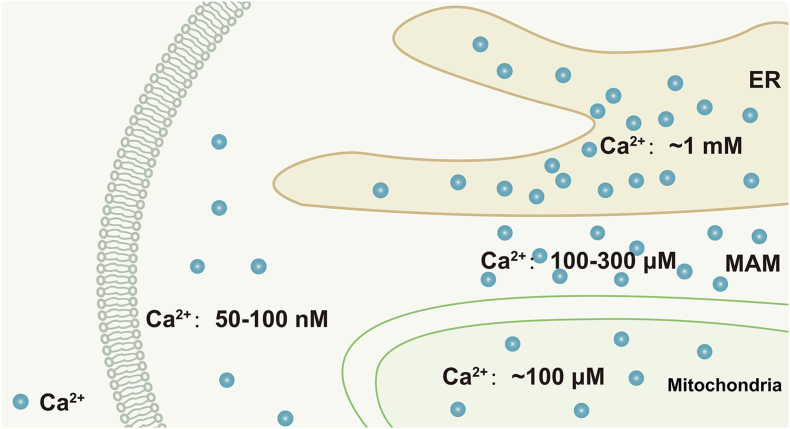
A schematic diagram of calcium ion concentrations in the cytoplasm, endoplasmic reticulum, and mitochondrial membranes, showing the differences in Ca^2+^ concentrations in each compartment. Created by the authors using Adobe Illustrator.

As the primary intracellular calcium reservoir, the ER sequesters the majority of cellular calcium. Upon release, most ER-derived Ca^2+^ is recycled via the sarcoplasmic/endoplasmic reticulum (SR/ER) Ca^2+^ ATPase (SERCA) pumps, a specific fraction is channeled specifically to mitochondria via the MAM interface [58]. Notably, the Ca^2+^ concentration within the MAM microdomain is significantly elevated compared to the cytoplasm. This gradient is essential for facilitating the rapid transfer of Ca^2+^ from the ER to the mitochondria, which is highly sensitive to Ca^2+^ fluctuation (Fig. 2) [59,60]. Yuan et al. discovered that the GRP75 protein present in MAM directly mediated the translocation of Ca^2+^ from the ER to the mitochondria [33]. As previously mentioned, the IP3R is the principal Ca^2+^ release channel. Li et al. reported that hypoxia condition induce the upregulation of IP3R at the MAM. This overexpression leads to an excessive influx of Ca^2+^, resulting in mitochondrial calcium overload and triggering cell death [61]. Beyond the conventional Ca^2+^ channels, Bidaux G. et al. identified a novel family of transient receptor potential melastatin 8 (TRPM8) isoforms that localized specifically to the MAM, which act as novel regulators of calcium homeostasis [62]. Thus, the intimate interconnection between the ER and mitochondria is fundamental for maintaining intracellular calcium homeostasis and supporting essential cellular activities.

#### 2.2.3 Cellular autophagy

Autophagy is a conserved cellular degradation process eliminating damaged organelles and proteins via the lysosomal pathway [63,64]. Under normal physiological conditions, this process typically induced by environmental stressors, such as nutrient deprivation or starvation, to recycle essential metabolic components. However, in t pathological conditions (including chronic inflammation and cancer), cells often exhibit dysregulated autophagic activity. The initiation of this complex process relies heavily on specific membrane architectures and typically proceeds within a specialized subdomain of the ER enriched in phosphatidylinositol 3-phosphate (PI3P). Within this compartment, PI3P plays a critical facilitating role by promoting the expression and localized aggregation of autophagy-related proteins [65]. This molecular assembly drives the biogenesis of phagocytic vesicles known as autophagosomes. As these vesicles expand, they progressively encapsulate the cargo slated for degradation, such as protein aggregates or damaged mitochondria. Subsequently, the autophagosomes mature and fuse with lysosomes to form autolysosomes, thereby completing the degradation process and enabling the enzymatic breakdown of the sequestered material.

Recent studies have established the MAM as a critical nucleation site and functional scaffold for autophagosome biogenesis, where essential autophagy proteins are recruited [66]. For example, Cheng X. et al. showed that under starvation conditions, the Soluble NSF Attachment Protein Receptor (SNARE) protein Syntaxin 17 (STX17) redistributes to the MAM. STX17 physically interacts with the PtdIns3 kinase subunit autophagy related 14 (ATG14) to promote autophagosome maturation [67]. Manganelli V. et al. reported specialized raft-like microdomains within the MAM that act as structural platforms for recruiting autophagosome proteins during the initiation phase [68].

The structural integrity of the MAM and the abundance of its resident proteins, particularly Mfn2, play a complex regulatory role in autophagy induced by external stressors such as metal toxicity or oxidative stress [69]. Wang X. Y. et al. showed that excessive exposure of Cu^2+^ disrupts MAM integrity, triggering a decrease in mitochondrial calcium levels and a downregulation of Mfn2 and VDAC1 expression. While overexpression of Mfn2 was found could alleviate this Cu-induced dysfunction, the study observed that restoring Mfn2 simultaneously enhanced the autophagic response in duck renal tubular epithelial cells. This finding suggests that Mfn2 contributes to maintain structural integrity while promoting stress-induced autophagy [70]. Similarly, under oxidative stress, Yang Y. T. et al. found that the knockdown of Mfn2 further exacerbates MAM dysregulation and mitochondrial dysfunction, whereas its overexpression enhances H_2_O_2_-induced MAM signaling. All these findings indicate that the MAM functions not only as the site of autophagosome formation but also as the membrane origin for oxidative stress-related autophagy, with Mfn2 acting as a key regulator in these adaptive responses [71].

### 2.3 The link between ER stress and mitochondrial stress

As previously discussed, the ER and mitochondria serve as central organelles responsible for cellular protein folding, lipid biosynthesis, and energy metabolism. These processes highly depend on specific homeostatic microenvironments. To ensure correct protein folding, the ER maintains a high redox potential via H_2_O_2_ generation and scavenging enzymes (e.g., GPX7 and GPX8), alongside a high calcium concentration sustained by SERCA pumps [72,73]. However, this precisely regulated environment is highly sensitive to disruption. Aberrant accumulation of ROS or calcium depletion impairs ER function, resulting in the aggregation of unfolded proteins and inducing ER stress [74,75]. Mitochondria, as the primary sites of ATP production, are vulnerable to stressors, such as chaperone depletion, oxidative damage, and genetic mutations. These factors induce mitochondrial stress, which is the accumulation of unfolded protein that initiates adaptive responses to restore organellar homeostasis.

In response to proteotoxic stress, both the ER and mitochondria activate dedicated stress-response pathways. The ER triggers the unfolded protein response (UPR^ER^) via transmembrane sensors, including Inositol-requiring enzyme 1 (IRE1α), PKR-like ER kinase (PERK), and Activating transcription factor 6 (ATF6). These pathways coordinately reduce global protein translation, enhance folding capacity, and promote degradation of misfolded proteins [76]. Meanwhile, mitochondria engage the mitochondrial unfolded protein response (UPR^mt^) under stress conditions [77]. In this pathway, stress impairs mitochondrial import of the transcription factor ATFS-1, preventing its degradation and allowing its accumulation in the nucleus, where it drives expression of mitochondrial chaperones and proteases [78,79]. In mammals, its homologous genes are ATF4 and ATF5, which perform the same functions. The activation of the UPR^ER^ and UPR^mt^ serves to preserve organelle homeostasis.

Several studies show that ER stress and mitochondrial stress are tightly interconnected via the integrated stress response (ISR), with the kinase PERK serving as a central bridge [80]. PERK is specifically enriched at ERMCS, and it inhibits global translation via eIF2α phosphorylation during ER stress [81]. Mitochondrial stress activates the metalloprotease OMA1, which cleaves DAP3 binding cell death enhancer 1 (DELE1). The cleaved DELE1 fragment accumulates in the cytosol, binds and activates the eIF2α kinase HRI, thereby triggering the ATF4–CHOP pathway. This pathway couples the UPR^mt^ to the ISR network, facilitating an integrated organellar stress responses [82,83]. Gao et al. demonstrated that pharmacological inhibition of the shared PERK/HRI–ATF4–CHOP axis suppresses stress-induced apoptosis and alleviated glaucomatous damage, underscoring the therapeutic potential of targeting ER–mitochondria stress signaling [84].

ER and mitochondria are functionally integrated through MAMs, which serve as hubs for Ca^2+^ transfer, lipid metabolism, autophagy, and stress signaling. The IP3R–GRP75–VDAC1 complex represents the central molecular module coupling ER Ca^2+^ release to mitochondrial uptake, thereby linking interorganellar communication to cell fate decisions. A major unresolved question is how MAM contacts are dynamically remodeled in response to physiological, metabolic, or oncogenic stress cues, and whether such remodeling can be pharmacologically targeted. Moreover, the dual pro-survival and pro-death roles of MAMs indicate that their therapeutic targeting requires a careful balance between preserving physiological organelle communication and inducing tumor-selective calcium overload. These concepts provide the mechanistic basis for understanding how calcium overload amplifies ER–mitochondria stress to drive tumor cell death.

## 3 Synergistic effects of calcium overload and ER–mitochondria stress

### 3.1 Calcium overload: the initial trigger that breaks homeostatic balance

Since calcium serves as a key regulator for oxidative phosphorylation - the primary pathway for ATP generation - intracellular Ca^2+^ concentration directly influences cellular bioenergetic efficiency and cell growth, both excessive and deficient Ca^2+^ levels can impair biological functions [85]. For instance, Fink et al. demonstrated that ATP production peaks when Ca^2+^ reaches 450 nM during respiration. Below this value, ATP synthesis rates decline, confirming that insufficient Ca^2+^ inhibits cellular energy production [86]. While physiological calcium levels support life, excessively high Ca^2+^ level would cause disruptive effects. Excessive Ca^2+^ accumulation initiates mitochondrial stress and triggers apoptotic pathways [87]. When mitochondrial Ca^2+^ accumulation exceeds the physiological buffering capacity, it can impair electron transport chain activity, suppress OXPHOS, reduce ADP-stimulated ATP production, and promote ROS generation. Moreover, massive mitochondrial Ca^2+^ accumulation triggers the opening of the mPTP in the inner mitochondrial membrane, leading to mitochondrial depolarization and bioenergetic failure [88,89]. Subsequently, proapoptotic proteins, such as cyt *c* and apoptosis-inducing factors, are released into the cytoplasm, leading to cell death [90]. This process, whereby aberrant cytoplasmic Ca^2+^ accumulation leads to tumor cell death, is defined as the “calcium overload strategy".

The central principle of calcium overload therapy is disrupting the calcium homeostasis of tumor cells to activate lethal signaling cascade. Current studies focus on developing diverse strategies to induce irreversible intracellular calcium accumulation. Based on the source of calcium ions, these strategies include exogenous calcium delivery, endogenous calcium release, and combinatorial approaches that synergize with other therapeutic modalities to induce calcium overload-driven synergistic therapeutic effects (Table 1).

**Table 1:** The strategy of calcium overload therapy for anti-tumor studies.

Strategy	Pathway	Mechanism	References
**Exogenous Ca^2+^ Delivery**	Calcium-based nanomaterials such as CaO_2_, CaCO_3_, etc.	Acidic TME triggers the decomposition of calcium-based nanomaterials to release Ca^2+^ and product H_2_O_2_inducing oxidative stress	[7,8]
**Endogenous Ca^2+^ Release**	Activation of ER Ca^2+^ release channels	Activating IP_3_R to trigger Ca^2+^ release from ER, disrupting intracellular Ca^2+^ homeostasis.	[[91], [92], [93]]
**Multichannel Synergistic Regulation**	Nanosystem simultaneously regulates endogenous and exogenous Ca^2+^	Inhibiting Ca^2+^ efflux, taking up exogenous Ca^2+^, or stimulating ER to release endogenous Ca^2+^ to disrupt intracellular Ca^2+^ homeostasis.	[94,95]
**Synergistic Combination Therapy**	Combined with PTT	Photothermal energy activates Ca^2+^ channels (e.g., TRPV1), destroys organelles, and inhibits Ca^2+^ efflux pumps, inducing Ca^2+^ elevation; Ca^2+^ overload and thermal damage synergistically kill tumor cells.	[9,10]
	Combined with SDT	Decomposition of CaO_2_ to induce Ca^2+^ overload, releases O_2_ to relieve hypoxia, and consumes GSH to enhance SDT sensitivity; sonosensitizers (e.g., Ce6) produce ROS under ultrasound, synergistically disrupting mitochondrial function.	[96,97]
	Combined with Gas Therapy	Gas molecules (e.g., NO) act as signaling molecules to regulate homeostasis by activating ER RyR channels via S-nitrosylation, opening TRPA1 channels, and modulating ATP supply.	[98,99]

### 3.2 Ca^2+^ collaborates with the crosstalk between ER and mitochondria to promote apoptosis in tumor cells

Intracellular Ca^2+^ overload results from homeostatic disruption caused by membrane damage, organelle dysfunction, or aberrant signaling. These processes are frequently associated with the dysregulated opening of membrane proteins, including voltage-gated calcium channels (VGCC), store-operated Ca^2+^ entry (SOCE) proteins (ORAI), and transient receptor potential (TRP) channels [100]. Upon entering the cytoplasm, Ca^2+^ is translocated into the mitochondria via VDAC) and the mitochondrial calcium uniporter (MCU) [101,102].

**Fig. 3:**
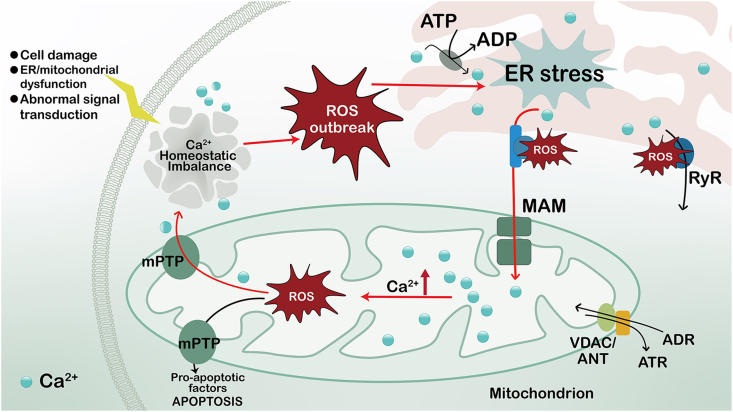
Synergistic effects of calcium overload and ER-mitochondrial stress. It shows the amplification between intracellular Ca^2+^ dysregulation and ER-mitochondria dysfunction, leading to mitochondrial permeability transition pore opening, oxidative stress, and apoptotic signaling. Created by the authors using Adobe Illustrator.

High concentrations of mitochondrial Ca^2+^ cause conformational changes in respiratory chain complexes. This leads to electron transport abnormalities and mitochondrial OXPHOS dysfunction, which further promote the generation of ROS [103]. The ROS diffuse into the cytoplasm and target ER channels like IP3R and ryanodine receptors (RyR), triggering the release of ER Ca^2+^ through MAMs, which intensifies mitochondrial calcium overload. ROS and ER Ca^2+^ depletion induce ER stress, further promoting Ca^2+^ influx into mitochondria via MAM-localized IP_3_R, establishing a vicious cycle [104]. Excessive mitochondrial Ca^2+^ and ROS interfere with the respiratory chain, deplete the inner membrane proton gradient, and induce mitochondrial stress and the UPR^mt^ [105]. Calcium overload, mitochondrial stress, and ER stress act synergistically to amplify ROS generation and cell damage (Fig. 3).

**Fig. 4:**
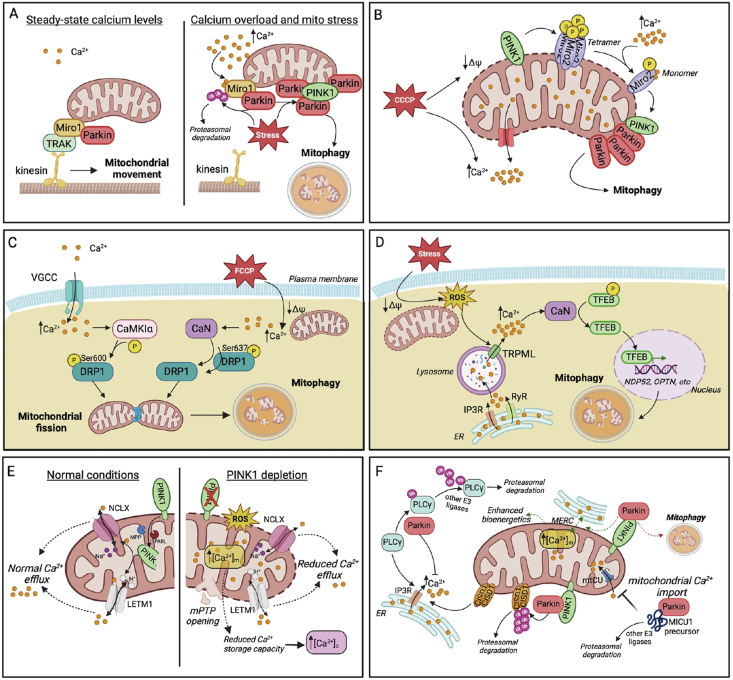
Calcium homeostasis impacts mitochondrial stress. (A) Low Ca^2+^ promotes mitochondrial movement; high Ca^2+^ induces mitophagy. (B) Mitochondrial depolarization triggers the accumulation of PINK1 and promotes Ca^2+^-dependent disassembly of Miro2/RHOT2, followed by the recruitment of Parkin (PINK1: PTEN-induced kinase 1, a mitochondrial damage sensor; Miro2/RHOT2: mitochondrial Rho GTPase 2/Ras homolog family member T2, an outer mitochondrial membrane GTPase controlling mitochondrial trafficking and Ca^2+^-dependent motility; Parkin: an E3 ubiquitin ligase promoting ubiquitin-dependent mitophagy). (C) Ca^2+^ accumulation activates fission proteins for mitochondrial fragmentation. (D) Mitochondrial ROS stimulate lysosomal Ca^2+^ release and mitophagy adaptor expression. (E) PINK1 phosphorylates calcium exchangers to prevent overload; its loss causes Ca^2+^ overload, ROS, and mPTP opening. (F) Parkin regulates calcium homeostasis, partly via ubiquitination of mitochondrial calcium uniporter regulators. Copyright permission from Ref. [106], 2025, Springer Nature. (For interpretation of the references to color in this figure legend, the reader is referred to the Web version of this article.)

Persistent calcium overload induces the sustained opening of the mPTP, causing membrane depolarization (Fig. 4) [106], loss of membrane potential (ΔΨm), and reduced ATP levels, ultimately leading to mitochondrial swelling, outer membrane rupture, and the release of cyt *c* to trigger apoptosis [107]. While inhibiting mPTP opening helps preserve cellular viability, its prolonged activation can also initiate the UPR^mt^ as an adaptive mechanism to maintain mitochondrial homeostasis [108,109]. However, an excessive UPR^mt^ may cause overexpression of associated genes, leading to mitochondrial protein insufficiency, oxidative stress, and functional impairment, which in turn promote apoptosis. Toll-like receptor (TLR) signaling activates UPRᵐᵗ via ROS and Ca^2+^ flux, inducing apoptosis through mitochondrial fragmentation and activation of the caspase-9/caspase-3 axis [110,111].

At the interface of organelles, excessive ROS released via the mPTP stimulate NADPH oxidase (NOX), promoting ROS production in ER. This oxidative stress, combined with Ca^2+^ depletion, triggers ER stress activates UPR^ER^ [60,112]. The PERK pathway plays a key role by phosphorylating eIF2α to upregulate ATF4 and CHOP, inhibiting Bcl-2 and assisting in the inter-organelle transfer of ROS and Ca^2+^ within MAMs, forming a positive feedback loop [113]. UPR^mt^ can also upregulate Bax via the eIF2α-ATF5 axis [114]; meanwhile, mitochondrial dysfunction leads to nuclear translocation of transcription factor ATFS-1 (Stress activated transcription factor) and accumulation of harmful mitochondrial DNA (AmtDNA), further impairing oxidative phosphorylation [115,116]. The bidirectional flow of ROS and Ca^2+^ at MAMs intensifies mitochondrial damage and mPTP opening, inducing tumor cell apoptosis through synergistic mechanisms [117]. Thus, this self-reinforcing calcium-ROS-stress cycle validates calcium overload as a potent strategy for effective tumor therapy (shown in Table 2).

**Table 2:** Calcium overload combined with ER-mitochondria stress in antitumor therapy.

Categories	Mechanism	Reference
Breast Cancer	The nanosystem disrupts mitochondria via SDT and delivers Ca^2+^, causing calcium overload that activates caspase-3/GSDME to induce pyroptosis.	[118]
	ROS and Ca^2+^ cause sequential mitochondrial and ER damage, activating the PERK-mediated elF2α phosphorylation pathway to induce immunogenic cell death.	[119]
	Oxygen/GSH depletion-boosted PDT destroys mitochondrial Ca^2+^ buffering capacity, causing Ca^2+^-overloading that inhibits ATP production for synergistic tumor therapy.	[120]
	The cascade amplification of ER stress and mitochondrial dysfunction initiated a cuproptosis, paraptosis, and apoptosis synergistic cell death pathway.	[121]
	Dual-enzyme-instructed peptide self-assembly coordinates TRPV1-mediated Ca^2+^ overload and chemotherapy to induce immunogenic cell death and elicit systemic adaptive immunity.	[122]
	Cannabinoids induce cell paraptosis by augmenting the Ca^2+^ flux between the ER and mitochondria, ultimately culminating in cell death.	[123]
	*Maclura pomifera* extract induces mitochondrial and ER stress through TRPV1 triggering intracellular calcium overload, initiating apoptosis via multiple pathways.	[124]
Lung Cancer	Kaempferol (KAE) disrupts calcium homeostasis and promotes calcium influx, while released calcium ions aggravate calcium overload, leading to apoptosis.	[125]
	Ultrasound activates Ti_3_C_2_/TiO_2_@CaCO_3_/KAE, dissolving CaCO_3_, switching nanosonosensitizer to “on” ROS state, exacerbating mitochondrial calcium overload, rapid Ca^2+^ release, inhibiting Ca^2+^ efflux.	[126]
Colon Cancer	Three-mode ROS outbreak generation via Fenton/Fenton-like reaction, consumption of glutathione, amelioration of hypoxia damages mitochondria, inhibits Ca^2+^ efflux channels, causing calcium overload that further increases ROS levels.	[127]
	pH-triggered dissociation, ultrasound-activated ROS generation, calcium overload leading to mitochondrial dysfunction, ER stress, STING/IRF3 pathway activation, and pyroptosis.	[128]
	Vanadate inhibits ATPases, induces potassium efflux and calcium overload, resulting in inflammasome activation, mitochondrial damage, while manganese ions stimulate STING pathway for robust pyroptosis and anti-tumor immunity.	[129]
Cervical Cancer	NIR light mediates Fe^3+^-to-Fe^2+^ reduction, activates photoacid generator, enabling Fenton reactions producing hydroxyl radicals while inducing calcium influx, leading to mitochondria calcium overload.	[130]
	N-pGE11 produces EGFR-specific nanofibers on cell membranes in ALP-responsive manner, facilitates extracellular Ca^2+^ influx, cytoplasmic Ca^2+^ overload, mitochondrial stress, caspase-3-dependent apoptosis, and thorough cell calcification.	[131]
Liver Cancer	CaCO_3_ nanoparticles released TAPP and Ca^2+^ in acidic environment, TAPP generating ^1^O_2_ under ultrasound, CuS produced ·OH in response to H_2_O_2_, causing mitochondrial damage and synergistic calcium overload with ROS therapy.	[132]
	Acid-activated Ca^2+^ release; photothermal TRPV1 activation increases Ca^2+^ influx; Fe-PDA reacts with H_2_O_2_ to produce ROS, inhibiting Ca^2+^ efflux pump causing overload.	[133]
Thyroid Cancer	Capsaicin activates the TRPV1 channel, induces intracellular mitochondrial calcium overload, leads to mitochondrial dysfunction, activates the mPTP channel, and ultimately results in apoptosis.	[134]
Ovarian Cancer	DOX-induced ROS elevation promotes ER stress and calcium homeostasis imbalance; Ca^2+^ accumulation aggravates mitochondrial dysfunction and triggers apoptotic cell death.	[135]
	Stigmasterol causes cancer cells to produce ROS and experience calcium overload, and stimulates cell death by activating the mitochondrial-ER stress axis.	[136]
Nasopharyngeal Carcinoma	Reticulocalbin (RCN2) promotes calcium overload by inducing ER stress to release Ca^2+^, which in turn triggers mitochondrial stress and mediates the activation of the mitochondrial apoptosis pathway.	[137]
Choriocarcinoma	The synergistic effect of apomorphine and paclitaxel induces ER stress, mitochondrial depolarization, calcium overload, energy deprivation, and apoptosis that is dependent on mitochondrial dysfunction.	[138]
Esophageal Cancer	IFI6 ablation induces mitochondrial calcium overload, activates mitochondrial Ca^2+^ uniporter, causes ROS production, suppresses supercomplex assembly, disrupts oxidative phosphorylation, drives ER stress via disrupted calcium uptake, upregulates NOX4-derived ROS.	[139]

Although Table 2 and the preceding sections summarize various calcium-based nanomaterials, their translational potential differs substantially in targeting efficiency, biocompatibility, and in vivo degradation behavior. CaCO_3_ nanoparticles generally show favorable biocompatibility and pH-responsive degradation in the acidic TME, but their tumor accumulation mainly depends on the EPR effect, which is heterogeneous and often limited in clinical tumors [[140], [141], [142]]. CaO_2_ nanoparticles can generate H_2_O_2_ to enhance oxidative stress in tumors; however, their high aqueous reactivity may reduce colloidal stability and circulation persistence and increase the risk of off-target oxidative injury [143,144]. Calcium phosphate (CaP) nanocarriers are composed of physiologically relevant calcium and phosphate ions, but unmodified CaP nanoparticles may aggregate in serum and therefore often require surface stabilization or targeting modification [135]. To improve clinical translatability, targeting efficiency can be enhanced via tumor-specific ligands or TME-responsive coatings [94,131]; biocompatibility favors FDA-approved materials like CaCO_3_ and CaP with optimized particle size [145,146]; Most calcium-based nanosystems remain preclinical, facing hurdles in scalable manufacturing, batch consistency, long-term toxicity assessment, and regulatory pathways [147]. Despite these challenges, their TME-responsive behavior and favorable safety profiles support further development, especially combined with active targeting or PTT/SDT [148].

### 3.3 Tumor type-specific molecular mechanisms of calcium overload

Although calcium overload represents a universal pro-apoptotic stress signal, its molecular execution pathways exhibit considerable heterogeneity across tumor types. Understanding this tumor-type specificity is important for patient stratification and the development of precision calcium-overload-based therapies.

In breast cancer, ER-positive models have been associated with activation of the PERK–eIF2α–ATF4–CHOP axis, Bcl-2 suppression, and mitochondrial outer membrane permeabilization, whereas some triple-negative breast cancer models show TRPV1-mediated Ca^2+^ influx followed by calpain-dependent caspase-12 activation [119,123,124]. In lung cancer, non-small cell lung cancer (NSCLC) models have been reported to involve IP3R–GRP75–VDAC1-mediated ER–mitochondria Ca^2+^ transfer, contributing to mitochondrial dysfunction and ferroptosis-related injury, while small cell lung cancer (SCLC) models may involve STING–IRF3-associated pyroptotic signaling [[126], [127], [128]]. In hepatocellular carcinoma, elevated MCU expression can promote mitochondrial Ca^2+^ accumulation, mPTP opening, and cytochrome *c* release; however, Mfn2-dependent protective autophagy may partially counteract apoptosis, suggesting that autophagy inhibition may enhance therapeutic efficacy [69,132]. In colorectal cancer, calcium overload can cooperate with ROS to induce GSDME-mediated pyroptosis, and p53-mutant contexts may further increase mitochondrial sensitivity to mPTP opening [118,128]. Other tumor types also show specialized responses: disturbed ER–mitochondria lipid metabolism has been linked to paraptosis-like death in ovarian cancer, RCN2 downregulation may facilitate IP3R-mediated apoptosis in nasopharyngeal carcinoma, and glioblastoma models have been associated with activation of the OMA1–DELE1–HRI mitochondrial stress pathway [83,84,[135], [136], [137]]. Tumor-type specificity in calcium overload arises from differences in upstream calcium influx, ER–mitochondria calcium transfer, mitochondrial Ca^2+^ handling, and downstream death-execution pathways. These mechanistic differences support the development of tumor-specific calcium-overload-based therapeutic strategies rather than a uniform approach across all cancers.

Calcium overload can convert physiological ER-mitochondria crosstalk into a self-amplifying death cascade. Excessive mitochondrial Ca^2+^ influx triggers mPTP opening, excessive ROS generation, and pro-apoptotic factor release, while ROS-dependent modulation of IP3R activity further exacerbates ER stress. Once initiated, this cycle can overwhelm the Ca^2+^-buffering capacity of tumor cells and trigger apoptosis, pyroptosis, or other regulated cell-death modalities. Importantly, the threshold for Ca^2+^-induced cell death varies markedly across tumor types since differences in Ca^2+^-handling machinery, such as MCU, IP3R, and TRPV1, as well as downstream death effectors. This tumor-type specificity suggests that universal calcium-overload strategies are unlikely to be equally effective across all cancers. Therefore, future calcium-overload strategies should consider not only apoptotic signaling but also non-apoptotic death modalities, including pyroptosis, paraptosis, and ferroptosis, which may contribute to tumor-type-specific therapeutic responses.

## 4 Future perspectives and challenges

The synergistic application of calcium overload and ER-mitochondria stress is a promising antitumor strategy, yet its clinical translation is limited by the risk of systemic toxicity arising from the calcium signaling in physiological homeostasis, and off-target calcium accumulation poses severe risks to excitable tissues [149].

### 4.1 Systemic toxicity and narrow therapeutic window

Calcium signaling is essential for normal physiological homeostasis, particularly in excitable tissues such as the heart and nervous system. Therefore, excessive exogenous Ca^2+^ delivery or uncontrolled endogenous Ca^2+^ release may cause severe adverse effects. In cardiomyocytes, Ca^2+^ overload can induce mPTP opening, mitochondrial depolarization, arrhythmias, contractile dysfunction, and even heart failure [150,151]. In neurons, aberrant Ca^2+^ accumulation may disrupt synaptic transmission, promote oxidative stress, and contribute to cognitive impairment or neurodegenerative injury [152]. Furthermore, abnormal calcium accumulation may affect hepatic and renal function by inducing hepatocellular injury, tubular epithelial damage, metabolic dysregulation, and cytoskeletal disruption [153]. According to the National Academy of Medicine, the tolerable upper intake level for calcium is 2500 mg/day for adults aged 19–50 years and 2000 mg/day for adults older than 50 years, considering total calcium intake from both diet and supplements [154].

To minimize systemic toxicity, calcium-overload-based therapies should achieve precise dose control and spatiotemporal regulation. Potential solutions include tumor-selective activation, local delivery, externally triggered release, real-time safety monitoring, and carefully optimized cardioprotective or neuroprotective interventions. Importantly, such protective strategies should be designed cautiously to avoid compromising the antitumor efficacy of calcium overload therapy.

### 4.2 Poor tumor specificity and off-target Ca^2+^ accumulation

Poor tumor specificity is another major obstacle, because off-target Ca^2+^ accumulation may damage normal tissues, especially excitable tissues. One promising strategy is to exploit the dysregulated calcium-handling machinery of cancer cells, including altered expression or activity of IP3R, TRPV1, MCU, and ORAI calcium channels. Small-molecule modulators, photosensitizers, or sonosensitizers may induce tumor-localized Ca^2+^ elevation by activating calcium channels or promoting endoplasmic reticulum/lysosomal Ca^2+^ release [148,[155], [156], [157]]. Another strategy employs TME-responsive nanocarriers that release Ca^2+^ only in the acidic or oxidative TME, sparing normal tissues [158]. However, current TME-responsive Ca^2+^-based nanoplatforms, such as calcium carbonate CaCO_3_, calcium peroxide CaO_2_, and calcium phosphate nanoparticles, often suffer from insufficient tumor accumulation, limited tumor retention, premature cargo leakage, and unpredictable biodistribution [159]. These limitations reduce therapeutic efficacy and increase the risk of systemic toxicity.

To address these issues, stimuli-responsive platforms triggered by acidic pH, ROS, GSH, or tumor-associated enzymes can be developed to improve tumor-selective Ca^2+^ release [158]. Active targeting strategies using tumor-specific ligands, such as hyaluronic acid, folate, peptides, aptamers, or antibodies, may further enhance tumor accumulation and cellular uptake [160,161]. Surface engineering, including biomimetic coatings or hydrophilic modification, may also improve circulation stability and tumor retention.

### 4.3 Nonspecific calcium release

Ca^2+^ signaling is highly dynamic, localized, and compartmentalized. Therefore, uncontrolled Ca^2+^ release may not only reduce antitumor efficacy but also increase side toxicity. Externally triggered therapeutic modalities, including photothermal therapy, photodynamic therapy, and sonodynamic therapy, offer spatially restricted activation and may enable more precise control of Ca^2+^ release within tumor tissues [9,10,117]. These approaches can also enhance tumor permeability, promote local nanocarrier retention, and synergistically amplify mitochondrial and ER stress.

In summary, although calcium overload provides a promising strategy for inducing tumor cell death, its clinical application is still constrained by insufficient tumor selectivity, potential toxicity to normal tissues, limited control of calcium release within tumors, and heterogeneity among different cancer types. Existing nanomedicine-based approaches, including ligand-mediated targeting, tumor microenvironment-responsive release, and externally assisted treatments such as PTT and SDT, have improved the feasibility of tumor-selective calcium intervention. However, these platforms should not be evaluated solely by their antitumor efficacy; their biocompatibility, biodegradation, organ distribution, pharmacokinetic behavior, and long-term safety are also important for translation. Given the tumor-type specificity discussed in Section 3.3, future studies should identify responsive tumor subtypes and relevant biomarkers to guide patient selection. Such a biology-guided design strategy may help transform calcium overload-based therapy from a preclinical concept into a more clinically relevant antitumor approach.

## 5 Conclusion

Intracellular calcium overload and ER-mitochondria stress are not isolated events but tightly interconnected processes that determine cell fate. Calcium dysregulation amplifies ER stress-induced apoptosis, while ER-mitochondria dysfunction further exacerbates calcium imbalance, creating a self-amplifying death cascade. This synergistic mechanism exerts potent anti-tumor effects by simultaneously affecting multiple survival pathways, thereby overwhelming the repair capacity of tumor cells and circumventing drug resistance. Despite the therapeutic potential, knowledge gaps still remain to fully explain the molecular mechanism. Future studies should focus on the explanation of molecular interaction networks linking calcium signaling to ER-mitochondria stress responses, this will facilitate the clinical translation of anticancer therapies that disrupt tumor-specific calcium homeostasis.

## Ethics approval

Not applicable.

## Declaration of generative AI in scientific writing

In this work, the AI tool (Deepseek) was used only for spelling checks.

## Funding information

This study was supported by Yantai Science and Technology Bureau (Grant No. 2024JCYJ063), Science and Technology Support Plan for Youth Innovation of Colleges and Universities of Shandong Province of China (Grant No.2022KJ344), National Natural Science Foundation of China (Grant No.82001961, 82473967, 82273969, 22377104), Innovative Drug Research and Development National Science and Technology Major Project (2025ZD1800504), Taishan Scholar Project (tsqn202211112).

## CRediT authorship contribution statement

**Yucui Ding:** Visualization, Writing – original draft. **Xinyu Liu:** Resources. **Jianyue Xue:** Resources. **Jianlong Fu:** Resources. **Sha Liu:** Resources. **Xiaopeng Xu:** Resources. **Xiang Jiao:** Resources. **Zhenyong Wu:** Resources. **Ganqiang Yang:** Resources. **Hongbo Wang:** Resources. **Peng Zhang:** Funding acquisition, Supervision, Writing – review & editing.

## Declaration of competing interest

The authors declare that they have no known competing financial interests or personal relationships that could have appeared to influence the work reported in this paper.

## Data Availability

No data was used for the research described in the article.

## References

[1] Wu Z.H., Xia F.N., Lin R. (2024). Global burden of cancer and associated risk factors in 204 countries and territories, 1980-2021: a systematic analysis for the GBD 2021. J. Hematol. Oncol..

[2] Wu P.J., Gao W., Su M., Nice E.C., Zhang W.H., Lin J., Xie N. (2021). Adaptive mechanisms of tumor therapy resistance driven by tumor microenvironment. Front. Cell Dev. Biol..

[3] Giannaccari M., Florindi C., Bloise N., Moccia F., Lodola F., Visai L. (2025). TRP channels and cancer modulation: a voyage beyond metabolic reprogramming, oxidative stress and the advent of nanotechnologies in targeted therapy. J. Exp. Clin. Cancer Res..

[4] Qin Z., Di Y., Ma T., Zeng W., Liu X., He W. (2025). The calcium homeostasis in tumor and the mechanism involving progression and metastasis. Cancer Lett..

[5] W. B. Zhao ,R. Sheng, The correlation between mitochondria-associated endoplasmic reticulum membranes (MAMs) and Ca(2+) transport in the pathogenesis of diseases, Acta Pharmacol. Sin. 46(2),1-21. 10.1038/S41401-024-01359-9. PMC11756407 39117969

[6] An G., Park J., Song J., Hong T., Song G., Lim W. (2024). Relevance of the endoplasmic reticulum-mitochondria axis in cancer diagnosis and therapy. Exp. Mol. Med..

[7] Liang J.L., Cao Y., Lv K., Xiao B., Sun J. (2025). Amplifying Ca(2+) overload by engineered biomaterials for synergistic cancer therapy. Biomaterials.

[8] Xie B., Dong L., Wang L., Wang R., Li C. (2024). Supramolecularly engineered bacteria mediated calcium overload and immunotherapy of tumors. Theranostics.

[9] Liu Z., Hu W., Cai Y., Wang N., Omer A.M., Ling J., Mei L., Ouyang X.K. (2025). Calcium peroxide functionalized mesoporous polydopamine nanoparticles triggered calcium overload for synergistic tumor gas/photothermal therapy. J. Colloid Interface Sci..

[10] Pang E., Li X., Zhao S., Tang Y., Xing X., Wang Q., Yang K., Wang B., Jin S., Song X., Lan M. (2024). Calcium-enriched carbon nanoparticles loaded with indocyanine green for near-infrared fluorescence imaging-guided synergistic calcium overload, photothermal therapy, and glutathione-depletion-enhanced photodynamic therapy. J. Mater. Chem. B.

[11] Pinton P. (2018). Mitochondria-associated membranes (MAMs) and pathologies. Cell Death Dis..

[12] Barazzuol L., Giamogante F., Calì T. (2021). Mitochondria associated membranes (MAMs): architecture and physiopathological role. Cell Calcium.

[13] Marchi S., Patergnani S., Pinton P. (2014). The endoplasmic reticulum-mitochondria connection: one touch, multiple functions. Biochim. Biophys. Acta.

[14] Liu Y., Qiao Y., Pan S., Chen J., Mao Z., Ren K., Yang Y., Feng Q., Liu D., Liu Z. (2023). Broadening horizons: the contribution of mitochondria-associated endoplasmic reticulum membrane (MAM) dysfunction in diabetic kidney disease. Int. J. Biol. Sci..

[15] Wang Y., Zhang Y., Jin Q., Zhao H., Li P. (2025). The role of mitochondrial-associated endoplasmic reticulum membranes (MAMs) in diabetic microvascular complications: a review. Cell Death Dis..

[16] Liu J., Yang J. (2022). Mitochondria-associated membranes: a hub for neurodegenerative diseases. Biomed. Pharmacother..

[17] Lee A., Sung G., Shin S., Lee S.Y., Sim J., Nhung T.T.M., Nghi T.D., Park S.K., Sathieshkumar P.P., Kang I., Mun J.Y., Kim J.S., Rhee H.W., Park K.M., Kim K. (2024). OrthoID: profiling dynamic proteomes through time and space using mutually orthogonal chemical tools. Nat. Commun..

[18] Wang X., Wen Y., Dong J., Cao C., Yuan S. (2018). Systematic In-Depth proteomic analysis of mitochondria-associated endoplasmic reticulum membranes in mouse and human testes. Proteomics.

[19] Li M., Zhang Y., Yu G., Gu L., Zhu H., Feng S., Xiong X., Jian Z. (2024). Mitochondria-associated endoplasmic reticulum membranes tethering protein VAPB-PTPIP51 protects against ischemic stroke through inhibiting the activation of autophagy. CNS Neurosci. Ther..

[20] Basso V., Marchesan E., Peggion C., Chakraborty J., von Stockum S., Giacomello M., Ottolini D., Debattisti V., Caicci F., Tasca E., Pegoraro V., Angelini C., Antonini A., Bertoli A., Brini M., Ziviani E. (2018). Regulation of ER-mitochondria contacts by Parkin via Mfn2. Pharmacol. Res..

[21] Hong H., Guo Z., Ge J., Li H. (2020). Mitochondria-associated endoplasmic reticulum membranes in health and diseases. MedComm.

[22] Manganelli V., Longo A., Mattei V., Recalchi S., Riitano G., Caissutti D., Capozzi A., Sorice M., Misasi R., Garofalo T. (2021). Role of ERLINs in the control of cell fate through lipid rafts. Cells.

[23] D'Angelo D., Rizzuto R. (2023). The Mitochondrial Calcium Uniporter (MCU): molecular identity and role in human diseases. Biomolecules.

[24] Mallilankaraman K., Doonan P., Cárdenas C., Chandramoorthy H.C., Müller M., Miller R., Hoffman N.E., Gandhirajan R.K., Molgó J., Birnbaum M.J., Rothberg B.S., Mak D.O., Foskett J.K., Madesh M. (2012). MICU1 is an essential gatekeeper for MCU-mediated mitochondrial Ca(2+) uptake that regulates cell survival. Cell.

[25] Kaufman R.J., Scheuner D., Schröder M., Shen X., Lee K., Liu C.Y., Arnold S.M. (2002). The unfolded protein response in nutrient sensing and differentiation. Nat. Rev. Mol. Cell Biol..

[26] Paillard M., Csordás G., Szanda G., Golenár T., Debattisti V., Bartok A., Wang N., Moffat C., Seifert E.L., Spät A., Hajnóczky G. (2017). Tissue-specific mitochondrial decoding of Cytoplasmic Ca(2+) signals is controlled by the stoichiometry of MICU1/2 and MCU. Cell Rep..

[27] Giorgi C., Marchi S., Pinton P. (2018). The machineries, regulation and cellular functions of mitochondrial calcium. Nat. Rev. Mol. Cell Biol..

[28] Csordás G., Várnai P., Golenár T., Roy S., Purkins G., Schneider T.G., Balla T., Hajnóczky G. (2010). Imaging interorganelle contacts and local calcium dynamics at the ER-Mitochondrial interface. Mol. Cell..

[29] Giorgi C., Marchi S., Pinton P. (2018). The machineries, regulation and cellular functions of mitochondrial calcium. Nat. Rev. Mol. Cell Biol..

[30] Baker M.R., Fan G., Arige V., Yule D.I., Serysheva I.I. (2023). Understanding IP(3)R channels: from structural underpinnings to ligand-dependent conformational landscape. Cell Calcium.

[31] Bartok A., Weaver D., Golenár T., Nichtova Z., Katona M., Bánsághi S., Alzayady K.J., Thomas V.K., Ando H., Mikoshiba K., Joseph S.K., Yule D.I., Csordás G., Hajnóczky G. (2019). IP(3) receptor isoforms differently regulate ER-mitochondrial contacts and local calcium transfer. Nat. Commun..

[32] Szabadkai G., Bianchi K., Várnai P., De Stefani D., Wieckowski M.R., Cavagna D., Nagy A.I., Balla T., Rizzuto R. (2006). Chaperone-mediated coupling of endoplasmic reticulum and mitochondrial Ca^2+^ channels. J. Cell Biol..

[33] Yuan M., Gong M., He J., Xie B., Zhang Z., Meng L., Tse G., Zhao Y., Bao Q., Zhang Y., Yuan M., Liu X., Luo C., Wang F., Li G., Liu T. (2022). IP3R1/GRP75/VDAC1 complex mediates endoplasmic reticulum stress-mitochondrial oxidative stress in diabetic atrial remodeling. Redox Biol..

[34] Liu Y., Ma X., Fujioka H., Liu J., Chen S., Zhu X. (2019). DJ-1 regulates the integrity and function of ER-mitochondria association through interaction with IP3R3-Grp75-VDAC1. Proc. Natl. Acad. Sci. USA.

[35] Basso V., Marchesan E., Ziviani E. (2020). A trio has turned into a quartet: DJ-1 interacts with the IP3R-Grp75-VDAC complex to control ER-mitochondria interaction. Cell Calcium.

[36] D'Eletto M., Rossin F., Occhigrossi L., Farrace M.G., Faccenda D., Desai R., Marchi S., Refolo G., Falasca L., Antonioli M., Ciccosanti F., Fimia G.M., Pinton P., Campanella M., Piacentini M. (2018). Transglutaminase type 2 regulates ER-Mitochondria contact sites by interacting with GRP75. Cell Rep..

[37] Li X.X., Zhao X., Qin Z.S., Li J., Sun B.W., Liu L. (2025). Regulation of calcium homeostasis in endoplasmic reticulum-mitochondria crosstalk: implications for skeletal muscle atrophy. Cell Commun. Signal..

[38] Zhou Y., Jing S.S., Liu S.N., Shen X.Z., Cai L.H., Zhu C.F., Zhao Y.C., Pang M.L. (2022). Double-activation of mitochondrial permeability transition pore opening via calcium overload and reactive oxygen species for cancer therapy. J. Nanobiotechnol..

[39] Waseem M., Wang B.D. (2023). Promising strategy of mPTP modulation in cancer therapy: an emerging progress and future Insight. Int. J. Mol. Sci..

[40] Xiao Y., Yang Y.X., Qiu H.S., Yang J., Liang H., Jiang Z.J., Tong X., Li Y.Y., Huang Q.F., Wu J.M., Lin T., Yu J., Liang M. (2026). CD44-Targeting hydroxyapatite nanoparticles (HAP) induce mitochondrial dysfunction-driven PANoptosis and Immunogenic Cell Death (ICD) via ca overload in colorectal cancer. Adv. Sci..

[41] Bravo-Sagua R., Torrealba N., Paredes F., Morales P.E., Pennanen C., López-Crisosto C., Troncoso R., Criollo A., Chiong M., Hill J.A., Simmen T., Quest A.F., Lavandero S. (2014). Organelle communication: signaling crossroads between homeostasis and disease. Int. J. Biochem. Cell Biol..

[42] Yang M., Li C., Yang S., Xiao Y., Xiong X., Chen W., Zhao H., Zhang Q., Han Y., Sun L. (2020). Mitochondria-associated ER membranes - the origin site of autophagy. Front. Cell Dev. Biol..

[43] Namba T., Tian F., Chu K., Hwang S.Y., Yoon K.W., Byun S., Hiraki M., Mandinova A., Lee S.W. (2013). CDIP1-BAP31 complex transduces apoptotic signals from endoplasmic reticulum to mitochondria under endoplasmic reticulum stress. Cell Rep..

[44] Inukai R., Mori K., Maki M., Takahara T., Shibata H. (2024). Cytoprotective role of autophagy in CDIP1 expression-induced apoptosis in MCF-7 breast cancer cells. Int. J. Mol. Sci..

[45] Iwasawa R., Mahul-Mellier A.L., Datler C., Pazarentzos E., Grimm S. (2011). Fis1 and Bap31 bridge the mitochondria-ER interface to establish a platform for apoptosis induction. EMBO J..

[46] Egner J.M., Nolden K.A., Harwig M.C., Bonate R.P., De Anda J., Tessmer M.H., Noey E.L., Ihenacho U.K., Liu Z., Peterson F.C., Wong G.C.L., Widlansky M.E., Hill R.B. (2022). Structural studies of human fission protein FIS1 reveal a dynamic region important for GTPase DRP1 recruitment and mitochondrial fission. J. Biol. Chem..

[47] Prinz W.A., Toulmay A., Balla T. (2020). The functional universe of membrane contact sites. Nat. Rev. Mol. Cell Biol..

[48] Vance J.E. (2014). MAM (Mitochondria-associated membranes) in mammalian cells: lipids and beyond. Biochim. Biophys. Acta.

[49] Zhong S., Li L., Liang N., Zhang L., Xu X., Chen S., Yin H. (2021). Acetaldehyde dehydrogenase 2 regulates HMG-CoA reductase stability and cholesterol synthesis in the liver. Redox Biol..

[50] Gonzalez-Baro M.R., Coleman R.A. (2017). Mitochondrial acyltransferases and glycerophospholipid metabolism. Biochim. Biophys. Acta Mol. Cell Biol. Lipids.

[51] Laquel P., Ayciriex S., Doignon F., Camougrand N., Fougère L., Rocher C., Wattelet-Boyer V., Bessoule J.J., Testet E. (2024). Mlg1, a yeast acyltransferase located in ER membranes associated with mitochondria (MAMs), is involved in de novo synthesis and remodelling of phospholipids. FEBS J..

[52] Chung K.P., Hsu C.L., Fan L.C., Huang Z., Bhatia D., Chen Y.J., Hisata S., Cho S.J., Nakahira K., Imamura M., Choi M.E., Yu C.J., Cloonan S.M., Choi A.M.K. (2019). Mitofusins regulate lipid metabolism to mediate the development of lung fibrosis. Nat. Commun..

[53] Petrungaro C., Kornmann B. (2019). Lipid exchange at ER-mitochondria contact sites: a puzzle falling into place with quite a few pieces missing. Curr. Opin. Cell Biol..

[54] Bionda C., Portoukalian J., Schmitt D., Rodriguez-Lafrasse C., Ardail D. (2004). Subcellular compartmentalization of ceramide metabolism: MAM (Mitochondria-associated membrane) and/or mitochondria?. Biochem. J..

[55] Zheng W., Pu M., Zeng S., Zhang H., Wang Q., Chen T., Zhou T., Chang C., Neculai D., Liu W. (2025). S-palmitoylation modulates ATG2-dependent non-vesicular lipid transport during starvation-induced autophagy. EMBO J..

[56] Li X., Yang Y., Shi X., Zhang Z., Ding S. (2024). Mitochondria-associated membranes as key regulators in cellular homeostasis and the potential impact of exercise on insulin resistance. Int. J. Mol. Sci..

[57] Marriott K.S., Prasad M., Thapliyal V., Bose H.S. (2012). σ-1 receptor at the mitochondrial-associated endoplasmic reticulum membrane is responsible for mitochondrial metabolic regulation. J. Pharmacol. Exp. Therapeut..

[58] Woll K.A., Van Petegem F. (2022). Calcium-release channels: structure and function of IP(3) receptors and ryanodine receptors. Physiol. Rev..

[59] Somlyo A.P., Bond M., Somlyo A.V. (1985). Calcium content of mitochondria and endoplasmic reticulum in liver frozen rapidly in vivo. Nature.

[60] Görlach A., Bertram K., Hudecova S., Krizanova O. (2015). Calcium and ROS: a mutual interplay. Redox Biol..

[61] Xu L., Xu Y., Jiang Y., Jiang J., Chen S., Sun D., Li S., Wei F., Zhu H. (2024). IP3R2 regulates apoptosis by Ca^2+^ transfer through mitochondria-ER contacts in hypoxic photoreceptor injury. Exp. Eye Res..

[62] Bidaux G., Gordienko D., Shapovalov G., Farfariello V., Borowiec A.S., Iamshanova O., Lemonnier L., Gueguinou M., Guibon R., Fromont G., Paillard M., Gouriou Y., Chouabe C., Dewailly E., Gkika D., López-Alvarado P., Carlos Menéndez J., Héliot L., Slomianny C., Prevarskaya N. (2018). 4TM-TRPM8 channels are new gatekeepers of the ER-mitochondria Ca(2+) transfer. Biochim. Biophys. Acta Mol. Cell Res..

[63] Mizushima N., Komatsu M. (2011). Autophagy: renovation of cells and tissues. Cell.

[64] Xu Y., Wu Y., Wang L., Ren Z., Song L., Zhang H., Qian C., Wang Q., He Z., Wan W. (2022). Autophagy deficiency activates rDNA transcription. Autophagy.

[65] Sciarretta S., Maejima Y., Zablocki D., Sadoshima J. (2018). The role of autophagy in the heart. Annu. Rev. Physiol..

[66] Qi L.F., Liu Y., Liu S., Xiang L., Liu Z., Liu Q., Zhao J.Q., Xu X. (2024). Phillyrin promotes autophagosome formation in A53T-αSyn-induced Parkinson's disease model via modulation of REEP1. Phytomedicine.

[67] Cheng X., Ma X., Ding X., Li L., Jiang X., Shen Z., Chen S., Liu W., Gong W., Sun Q. (2017). Pacer mediates the function of class III PI3K and HOPS complexes in autophagosome maturation by engaging Stx17. Mol. Cell.

[68] Manganelli V., Matarrese P., Antonioli M., Gambardella L., Vescovo T., Gretzmeier C., Longo A., Capozzi A., Recalchi S., Riitano G., Misasi R., Dengjel J., Malorni W., Fimia G.M., Sorice M., Garofalo T. (2021). Raft-like lipid microdomains drive autophagy initiation via AMBRA1-ERLIN1 molecular association within MAMs. Autophagy.

[69] Li H., Dong A., Liu C., He P., Ma Y., Chen S., Dong S., Zhang S., Zhang M., Zhang M. (2025). Schisandra chinensis mixture attenuates diabetic kidney disease via VDAC1/Grp75/IP(3)R-mediated MAMs stabilization and apoptosis-autophagy regulation. Phytomedicine.

[70] Wang X., Cao H., Fang Y., Bai H., Chen J., Xing C., Zhuang Y., Guo X., Hu G., Yang F. (2022). Activation of endoplasmic reticulum-mitochondria coupling drives copper-induced autophagy in duck renal tubular epithelial cells. Ecotoxicol. Environ. Saf..

[71] Yang Y., Wu J., Lu W., Dai Y., Zhang Y., Sun X. (2023). Mitochondria-associated endoplasmic reticulum membranes dysfunction contributes to PARP-1-dependent cell death under oxidative stress in retinal precursor cells. J. Biochem. Mol. Toxicol..

[72] Duan J., Zhang T., Gaffrey M.J., Weitz K.K., Moore R.J., Li X., Xian M., Thrall B.D., Qian W.J. (2020). Stochiometric quantification of the thiol redox proteome of macrophages reveals subcellular compartmentalization and susceptibility to oxidative perturbations. Redox Biol..

[73] Yoboue E.D., Rimessi A., Anelli T., Pinton P., Sitia R. (2017). Regulation of calcium fluxes by GPX8, a Type-II transmembrane peroxidase enriched at the mitochondria-associated endoplasmic reticulum membrane. Antioxid Redox Signal.

[74] Chaudhari N., Talwar P., Parimisetty A., Lefebvre d'Hellencourt C., Ravanan P. (2014). A molecular web: endoplasmic reticulum stress, inflammation, and oxidative stress. Front. Cell. Neurosci..

[75] Görlach A., Klappa P., Kietzmann T. (2006). The endoplasmic reticulum: folding, calcium homeostasis, signaling, and redox control. Antioxid Redox Signal.

[76] Grootjans J., Kaser A., Kaufman R.J., Blumberg R.S. (2016). The unfolded protein response in immunity and inflammation. Nat. Rev. Immunol..

[77] Ryan M.T., Hoogenraad N.J. (2007). Mitochondrial-nuclear communications. Annu. Rev. Biochem..

[78] Quirós P.M., Mottis A., Auwerx J. (2016). Mitonuclear communication in homeostasis and stress. Nat. Rev. Mol. Cell Biol..

[79] Nargund A.M., Pellegrino M.W., Fiorese C.J., Baker B.M., Haynes C.M. (2012). Mitochondrial import efficiency of ATFS-1 regulates mitochondrial UPR activation. Science.

[80] Verfaillie T., Rubio N., Garg A.D., Bultynck G., Rizzuto R., Decuypere J.P., Piette J., Linehan C., Gupta S., Samali A., Agostinis P. (2012). PERK is required at the ER-mitochondrial contact sites to convey apoptosis after ROS-based ER stress. Cell Death Differ..

[81] Hetz C., Zhang K., Kaufman R.J. (2020). Mechanisms, regulation and functions of the unfolded protein response. Nat. Rev. Mol. Cell Biol..

[82] Fessler E., Eckl E.M., Schmitt S., Mancilla I.A., Meyer-Bender M.F., Hanf M., Philippou-Massier J., Krebs S., Zischka H., Jae L.T. (2020). A pathway coordinated by DELE1 relays mitochondrial stress to the cytosol. Nature.

[83] Guo X., Aviles G., Liu Y., Tian R., Unger B.A., Lin Y.T., Wiita A.P., Xu K., Correia M.A., Kampmann M. (2020). Mitochondrial stress is relayed to the cytosol by an OMA1-DELE1-HRI pathway. Nature.

[84] Gao Z., Li M., Yao F., Xia X., Duan T., Meng J., Huang Y., He Y., Saro A., Huang J. (2022). Valdecoxib protects against cell apoptosis induced by endoplasmic reticulum stress via the inhibition of PERK-ATF4-CHOP pathway in experimental glaucoma. Int. J. Mol. Sci..

[85] Bhosale G., Sharpe J.A., Sundier S.Y., Duchen M.R. (2015). Calcium signaling as a mediator of cell energy demand and a trigger to cell death. Ann. N. Y. Acad. Sci..

[86] Fink B.D., Bai F., Yu L., Sivitz W.I. (2017). Regulation of ATP production: dependence on calcium concentration and respiratory state. Am. J. Physiol. Cell Physiol..

[87] Guo X., Wang L., Xuan J., Chen T., Du Y., Qiao H., Zhang S., Sun Z., Wang J., Niu R. (2025). Fluoride induces spermatocyte apoptosis by IP3R1/MCU-mediated mitochondrial calcium overload through MAMs. J. Hazard Mater..

[88] Brookes P.S., Yoon Y.S., Robotham J.L., Anders M.W., Sheu S.S. (2004). Calcium, ATP, and ROS: a mitochondrial love-hate triangle. Am. J. Physiol. Cell Physiol..

[89] Gunter T.E., Sheu S.-S. (2009). Characteristics and possible functions of mitochondrial Ca^2+^ transport mechanisms. BBA Bioenerg..

[90] Bonora M., Giorgi C., Pinton P. (2022). Molecular mechanisms and consequences of mitochondrial permeability transition. Nat. Rev. Mol. Cell Biol..

[91] Ma Y., Zhang Z., Yu D., Sun G., Wang T., Yan H., Zhang Z., Liu J., Zhu D. (2025). A Snakeberry-Inspired photocatalytic nanoreactor activates endogenous Ca(2+) Store for Ca(2+) overload-mediated cancer therapy. Small.

[92] Zheng B.X., Long W., Zeng Y.X., She M.T., Zheng Y., Zheng W.D., Wang Y.K., Chan K.H., Leung A.S., Chan C.M., Lu Y.J., Wong W.L. (2025). A mitochondria-targeting and G-quadruplex structure-binding ligand inducing calcium overload and ferroptosis in human cancer cells. Br. J. Pharmacol..

[93] Sun P., Wang S., Wang H., Li Y., Wang S., Chen X., Wu Z., Qi X. (2025). Polymerized apoferritin: a promising carrier for efficient compartmentalized and deep-layer delivery of calcium donors in tumor ca overload therapy. J. Contr. Release.

[94] Li L., Xing Z., Wang J., Guo Y., Wu X., Ma Y., Xu Z., Kuang Y., Liao T., Li C. (2025). Hyaluronic acid-mediated targeted nano-modulators for activation of pyroptosis for cancer therapy through multichannel regulation of Ca(2+) overload. Int. J. Biol. Macromol..

[95] Chen X.A., Xu C., Zhao P., Zhang Y., Guo J.Z., Hu X.L., Gao H., Zhang C.N., Qu X.W., Zhang J.M. (2023). A multichannel Ca^2+^nanomodulator amplifies exogenous and endogenous calcium overload for efficient antitumor and antimetastasis therapy. Chem. Eng. J..

[96] Zhao Y., Zhang Y., Zhang Y., Zhang Y., Deng Z., Bai T., Zhang M., Zhang M., Song J. (2025). Biomimetic nanoplatform-mediated protective autophagy blockage enhancing sonodynamic and Ca(2+)-Overload combined therapy for Colon cancer. Small Methods.

[97] He Y., Yang X., Yuan M., Zhang X., Tu W., Xue W., Wang D., Gao D. (2025). Wireless discharge of piezoelectric nanogenerator opens voltage-gated ion channels for calcium overload-mediated tumor treatment. Biomaterials.

[98] Li Y., Pan Y., Chen C., Li Z., Du S., Luan X., Gao Y., Han X., Song Y. (2022). Multistage-responsive gene editing to sensitize ion-interference enhanced carbon monoxide gas therapy. Small.

[99] Ren B., Wang Y., Wang H.Y., Tang Q., Yang S.P., Liu J.G., Xiang H.J. (2025). Engineering dual-gas releasing nanoplatform for enhancing endogenous Ca^2+^-mediated ion interference therapy. Nano Today.

[100] Hu J.J., Yuan L., Zhang Y., Kuang J., Song W., Lou X., Xia F., Yoon J. (2024). Photo-controlled calcium overload from endogenous sources for tumor therapy. Angew Chem. Int. Ed. Engl..

[101] Marchi S., Giorgi C., Galluzzi L., Pinton P. (2020). Ca(2+) fluxes and cancer. Mol. Cell.

[102] Álvarez-Illera P., García-Casas P., Fonteriz R.I., Montero M., Alvarez J. (2020). Mitochondrial Ca(2+) dynamics in MCU knockout C. elegans worms. Int. J. Mol. Sci..

[103] Thiriveedi V.R., Mattam U., Pattabhi P., Bisoyi V., Talari N.K., Krishnamoorthy T., Sepuri N.B.V. (2020). Glutathionylated and Fe-S cluster containing hMIA40 (CHCHD4) regulates ROS and mitochondrial complex III and IV activities of the electron transport chain. Redox Biol..

[104] Li X., Liang M., Jiang J., He R., Wang M., Guo X., Shen M., Qin R. (2018). Combined inhibition of autophagy and Nrf2 signaling augments bortezomib-induced apoptosis by increasing ROS production and ER stress in pancreatic cancer cells. Int. J. Biol. Sci..

[105] Senese R., Petito G., Silvestri E., Ventriglia M., Mosca N., Potenza N., Russo A., Falvo S., Manfrevola F., Cobellis G., Chioccarelli T., Porreca V., Mele V.G., Chianese R., de Lange P., Ricci G., Cioffi F., Lanni A. (2024). The impact of cannabinoid receptor 1 absence on mouse liver mitochondria homeostasis: insight into mitochondrial unfolded protein response. Front. Cell Dev. Biol..

[106] Borbolis F., Ploumi C., Palikaras K. (2025). Calcium-mediated regulation of mitophagy: implications in neurodegenerative diseases. NPJ Metab. Health Dis..

[107] Zoratti M., Biasutto L., Parrasia S., Szabo I. (2024). Mitochondrial permeability transition pore: a snapshot of a therapeutic target. Expert Opin. Ther. Targets.

[108] Huang P., Wu S.P., Wang N., Seto S., Chang D. (2021). Hydroxysafflor yellow A alleviates cerebral ischemia reperfusion injury by suppressing apoptosis via mitochondrial permeability transition pore. Phytomedicine.

[109] Angeli S., Foulger A., Chamoli M., Peiris T.H., Gerencser A., Shahmirzadi A.A., Andersen J., Lithgow G. (2021). The mitochondrial permeability transition pore activates the mitochondrial unfolded protein response and promotes aging. eLife.

[110] Chen W.C., Zhang P., Ding Y.C., Xie X.B., Fu J.L., Zhao R., Xiao Y.H., Lukic M.J., Li B., Wang W.S., Chen S. (2024). Bioactives from biomass: treasure for future potent antimicrobial applications. Chem. Eng. J..

[111] Kumar M., Sharma S., Haque M., Kumar J., Hathi U.P.S., Mazumder S. (2022). TLR22-Induced pro-apoptotic mtROS abets UPR(mt)-Mediated mitochondrial fission in aeromonas hydrophila-Infected headkidney macrophages of Clarias gariepinus. Front. Immunol..

[112] Zhao S., Feng H., Jiang D., Yang K., Wang S.T., Zhang Y.X., Wang Y., Liu H., Guo C., Tang T.S. (2023). ER Ca(2+) overload activates the IRE1α signaling and promotes cell survival. Cell Biosci..

[113] Ludwig M.P., Galbraith M.D., Eduthan N.P., Hill A.A., Clay M.R., Tellez C.M., Wilky B.A., Elias A., Espinosa J.M., Sullivan K.D. (2023). Proteasome inhibition sensitizes liposarcoma to MDM2 inhibition with Nutlin-3 by activating the ATF4/CHOP stress response pathway. Cancer Res..

[114] Shen Z., Liu P., Sun Q., Li Y., Acharya R., Li X., Sun C. (2021). FTO inhibits UPR(mt)-induced apoptosis by activating JAK2/STAT3 pathway and reducing m6A level in adipocytes. Apoptosis.

[115] Lin Y.F., Schulz A.M., Pellegrino M.W., Lu Y., Shaham S., Haynes C.M. (2016). Maintenance and propagation of a deleterious mitochondrial genome by the mitochondrial unfolded protein response. Nature.

[116] Melber A., Haynes C.M. (2018). UPR(mt) regulation and output: a stress response mediated by mitochondrial-nuclear communication. Cell Res..

[117] Zhu P., Hu S., Jin Q., Li D., Tian F., Toan S., Li Y., Zhou H., Chen Y. (2018). Ripk3 promotes ER stress-induced necroptosis in cardiac IR injury: a mechanism involving calcium overload/XO/ROS/mPTP pathway. Redox Biol..

[118] Zhang Z., Zhang X., Zhao S.S., Dong C.H., Feng W., Yu L.D., Ding L., Chen Y., Chen B.D. (2023). Nanosonodynamic effect-promoted mitochondrial dysfunction augments calcium overload for gasdermin E-induced pyroptotic antitumor therapy. Chem. Eng. J..

[119] Feng X., Lin T., Chen D., Li Z., Yang Q., Tian H., Xiao Y., Lin M., Liang M., Guo W., Zhao P., Guo Z. (2023). Mitochondria-associated ER stress evokes immunogenic cell death through the ROS-PERK-eIF2α pathway under PTT/CDT combined therapy. Acta Biomater..

[120] Zhu J., Jiao A., Li Q., Lv X., Wang X., Song X., Li B., Zhang Y., Dong X. (2022). Mitochondrial Ca(2+)-overloading by oxygen/glutathione depletion-boosted photodynamic therapy based on a CaCO(3) nanoplatform for tumor synergistic therapy. Acta Biomater..

[121] Xu W., Suo A., Aldai A.J.M., Wang Y., Fan J., Xia Y., Xu J., Chen Z., Zhao H., Zhang M., Qian J. (2024). Hollow calcium/copper bimetallic amplifier for cuproptosis/paraptosis/apoptosis cancer therapy via Cascade reinforcement of endoplasmic reticulum stress and mitochondrial dysfunction. ACS Nano.

[122] Zhang Z.H., Hu Y.H., Ding Y.H., Zhang X.Y., Dong X., Xie L.M., Yang Z.M., Hu Z.W. (2025). Dual-enzyme-instructed peptide self-assembly to boost immunogenic cell death by coordinating intracellular calcium overload and chemotherapy. ACS Nano.

[123] de la Harpe A., Beukes N., Frost C. (2024). Mitochondrial calcium overload contributes to cannabinoid-induced paraptosis in hormone-responsive breast cancer cells. Cell Prolif..

[124] Rumpa M.M., Maier C. (2024). TRPV1-Dependent antiproliferative activity of dioecious Maclura pomifera extracts in estrogen receptor-positive breast cancer cell lines involves multiple apoptotic pathways. Int. J. Mol. Sci..

[125] Li Y., Zhou S., Song H., Yu T., Zheng X., Chu Q. (2021). CaCO(3) nanoparticles incorporated with KAE to enable amplified calcium overload cancer therapy. Biomaterials.

[126] Yu H., Huang Y., Cai Z., Huang K., Yu T., Lan H., Zhang Q., Wu L., Luo H. (2024). Tumor microenvironment-sensitive Ca(2+) nanomodulator combined with the sonodynamic process for enhanced cancer therapy. ACS Appl. Mater. Interfaces.

[127] Xu W., Zhou H., Hu B., Liang X., Tang Y., Ning S., Ding H., Yang P., Wang C. (2024). Prussian blue-derived nanocomposite synergized with calcium overload for three-mode ROS outbreak generation to enhance oncotherapy. Adv. Healthcare Mater..

[128] Chen Q., Peng B., Lin L., Chen J., Jiang Z., Luo Y., Huang L., Li J., Peng Y., Wu J., Li W., Zhuang K., Liang M. (2024). Chondroitin sulfate-modified hydroxyapatite for Caspase-1 activated induced pyroptosis through Ca Overload/ER Stress/STING/IRF3 pathway in colorectal cancer. Small.

[129] Pei Z.F., Jiang N., Gong F., Yang W.H., Xu J.C., Yu B., Yang N.L., Wu J., Lei H.L., Sun S.M., Li L.X., Liu Z.C., Ni C.F., Cheng L. (2024). A metal anion strategy to induce pyroptosis combined with STING activation to synergistically amplify anti-tumor immunity. Mater. Today.

[130] Bao W., Liu M., Meng J., Liu S., Wang S., Jia R., Wang Y., Ma G., Wei W., Tian Z. (2021). MOFs-based nanoagent enables dual mitochondrial damage in synergistic antitumor therapy via oxidative stress and calcium overload. Nat. Commun..

[131] Zhang Z.H., Sun X., Ding Y.H., Zhang X.Y., Zhang Y.M., Zhang T.X., Li J., Wang L., Yang Z.M., Hu Z.W. (2023). Membrane-bound supramolecular nanofiber network stimulates transient receptor potential channels for calcium overload cancer therapy. Nano Today.

[132] Zhang B., Man J., Guo L., Ru X., Zhang C., Liu W., Li L., Ma S., Guo L., Wang H., Wang B., Diao H., Che R., Yan L. (2024). Layer-by-Layer nanoparticles for calcium overload in situ enhanced reactive oxygen oncotherapy. Int. J. Nanomed..

[133] Zhao F., Wang C., Wang H., Ying Y., Li W., Li J., Zheng J., Qiao L., Che S., Yu J. (2024). Acidity-responsive Fe-PDA@CaCO(3) nanoparticles for photothermal-enhanced Calcium-Overload- and reactive-oxygen-species-mediated tumor therapy. ACS Appl. Mater. Interfaces.

[134] Xu S., Cheng X., Wu L., Zheng J., Wang X., Wu J., Yu H., Bao J., Zhang L. (2020). Capsaicin induces mitochondrial dysfunction and apoptosis in anaplastic thyroid carcinoma cells via TRPV1-mediated mitochondrial calcium overload. Cell. Signal..

[135] Qiu M., Chen J., Huang X., Li B., Zhang S., Liu P., Wang Q., Qian Z.R., Pan Y., Chen Y., Zhao J. (2022). Engineering chemotherapeutic-augmented calcium phosphate nanoparticles for treatment of intraperitoneal disseminated ovarian cancer. ACS Appl. Mater. Interfaces.

[136] Bae H., Song G., Lim W. (2020). Stigmasterol causes ovarian cancer cell apoptosis by inducing endoplasmic reticulum and mitochondrial dysfunction. Pharmaceutics.

[137] Yao H., Zhang S., Xie H., Fan Y., Miao M., Zhu R., Yuan L., Gu M., You Y., You B. (2023). RCN2 promotes nasopharyngeal carcinoma progression by curbing calcium flow and mitochondrial apoptosis. Cell. Oncol..

[138] Lee J.Y., Ham J., Lim W., Song G. (2020). Apomorphine induces mitochondrial-dysfunction-dependent apoptosis in choriocarcinoma. Reproduction.

[139] Liu Z., Gu S., Lu T., Wu K., Li L., Dong C., Zhou Y. (2020). IFI6 depletion inhibits esophageal squamous cell carcinoma progression through reactive oxygen species accumulation via mitochondrial dysfunction and endoplasmic reticulum stress. J. Exp. Clin. Cancer Res..

[140] Mosaheb M., Holl M.M.B., Tha K.K., Abidin S.A.Z., Chowdhury E.H. (2025). Fabrication and characterization of calcium carbonate nanoparticles for delivery of doxorubicin in breast cancer cells. J. Drug Deliv. Sci. Technol..

[141] Luo W.L., Li Z.J., Zhang L., Xie X.Y. (2023). Polyethylenimine-CO2 adduct templated CaCO_3_ nanoparticles as anticancer drug carrier. Cancer Nanotechnol..

[142] Lin C.T., Akhtar M., Li Y.J., Ji M., Huang R.Q. (2024). Recent developments in CaCO3 nano-drug delivery systems: advancing biomedicine in tumor diagnosis and treatment. Pharmaceutics.

[143] Zhang M., Song R.X., Liu Y.Y., Yi Z.G., Meng X.F., Zhang J.W., Tang Z.M., Yao Z.W., Liu Y., Liu X.G., Bu W.B. (2019). Calcium-overload-mediated tumor therapy by calcium peroxide nanoparticles. Chem.

[144] Alexis F., Pridgen E., Molnar L.K., Farokhzad O.C. (2008). Factors affecting the clearance and biodistribution of polymeric nanoparticles. Mol. Pharm..

[145] Deng X., Zanna M.Y., Ahmed H., Ismail N., Chan K.W., Esa N.M., Razis A.F.A., Bakar M.Z.A., Yang Z.M. (2025). Recent progress in calcium carbonate nanoparticles for cancer therapy: design strategies and clinical prospects. Mater. Res. Express.

[146] Qiu C., Wu Y.Y., Guo Q.Y., Shi Q.L., Zhang J.Z., Meng Y.Q., Xia F., Wang J.G. (2022). Preparation and application of calcium phosphate nanocarriers in drug delivery. Mater. Today Bio.

[147] Gangadhar L., Sana S.S., Venkatesan R., Bhandare R., Kim S.-C., Yasin H. (2026). Next generation calcium nanomaterials: disrupting tumor Ca^2+^ homeostasis for enhanced cancer therapy. Int. J. Nanomed..

[148] Wang C.L., Peng J.R., Xiao Y.W., Zhang Z.Q., Yang X., Liang X.Y., Yang J., Zhou X.Y., Li C.H. (2025). Advances in nanotherapeutics for tumor treatment by targeting calcium overload. Colloids Surf. B Biointerfaces.

[149] Vassalle M., Lin C.I. (2004). Calcium overload and cardiac function. J. Biomed. Sci..

[150] Malig T., Xiao Z., Chen S.R., Back T.G. (2016). Suppression of store overload-induced calcium release by hydroxylated metabolites of carvedilol. Bioorg. Med. Chem. Lett..

[151] Wang R., Wang M., Zhou J., Dai Z., Sun G., Sun X. (2021). Calenduloside E suppresses calcium overload by promoting the interaction between L-type calcium channels and Bcl2-associated athanogene 3 to alleviate myocardial ischemia/reperfusion injury. J. Adv. Res..

[152] Zhang H., Zhou H., Guo X., Zhang G., Xiao M., Wu S., Jin C., Yang J., Lu X. (2023). Cigarette smoke triggers calcium overload in mouse hippocampal neurons via the ΔFOSB-CACNA2D1 axis to impair cognitive performance. Ecotoxicol. Environ. Saf..

[153] Chen X., Hu K., Zhang Y., He S.M., Wang D.D. (2024). Targeting CXCR2 ameliorated tacrolimus-induced nephrotoxicity by alleviating overactivation of PI3K/AKT/mTOR pathway and calcium overload. Biomed. Pharmacother..

[154] Uusi-Rasi K., Kärkkäinen M.U.M., Lamberg-Allardt C.J.E. (2013). Calcium intake in health maintenance - a systematic review. Food Nutr. Res..

[155] Li Z.X., Ran Q., Qu C., Hu S., Cui S.Y., Zhou Y., Shen B., Yang B. (2025). Sigma-1 receptor activation attenuates DOX-induced cardiotoxicity by alleviating endoplasmic reticulum stress and mitochondrial calcium overload via PERK and IP3R-VDAC1-MCU signaling pathways. Biol. Direct.

[156] Singh A.K., Roy N.K., Bordoloi D., Padmavathi G., Banik K., Khwairakpam A.D., Kunnumakkara A.B., Sukumar P. (2020). Orai-1 and Orai-2 regulate oral cancer cell migration and colonisation by suppressing Akt/mTOR/NF-κB signalling. Life Sci..

[157] Ni X.Y., Shi W.H., Liu Y.X., Yin L.K., Guo Z.X., Zhou W., Fan Q.L. (2022). Capsaicin-decorated semiconducting polymer nanoparticles for light-controlled calcium-Overload/Photodynamic combination therapy. Small.

[158] Chen M., Wang Y., Niu Y.R., Chen X.M., Su H., Xia L., Liu C.B., Zhou J.F., Wang Z., Li B., Lu D.Y. (2026). pH-responsive CaCO_3_ nanoplatform amplifies SDT via calcium overload-ROS loop for deep tumor therapy. iScience.

[159] Dong J.B., Wen L.T., Zhang C., Chen Z.F., Gong Q.F., Song G.S., Liang C. (2026). Calcium-free nanomaterials-mediated calcium overloading for cancer therapy. Sci. China Mater..

[160] Tang Y.C., Huang J.R., Cui C., Yang F.Y., Li K.F., Gao B.J., Fu S.Z., Yang X.L. (2025). Folate-modified smart responsive nanosystems for enhancing anti-tumor therapy through calcium overload and chemotherapy. Int. J. Nanomed..

[161] Zhu X., Lu Y.T., Sun M.H., Wu X.D., Zhang H., Wang Z.Q., Jin Y.X., Tan G.H. (2025). A hyaluronic acid schiff base @ hollow calcium peroxide @ copper carbon dots nanoconjugate enhances chemodynamic tumor therapy based on multiple approaches strategy. Int. J. Biol. Macromol..

